# Hawaiian Laws for the Government of Cattle-Traffic

**Published:** 1898-03

**Authors:** 


### CONTROL WORK.

### TO PROVIDE FOR THE SUPPRESSION OF DISEASE AMONG ANIMALS IN THE HAWAIIAN ISLANDS.

SECTION 1. The Minister of the Interior is hereby authorized and directed to establish, on each of the islands of the Kingdom having ports of entry, a quarantine station or stations for animals.

SEC. 2. The Minister of the Interior is hereby authorized to appoint three competent persons for each port of entry of the Kingdom, who shall be designated "Board of Inspectors of Animals," and from time to time, when such offices, for any reason, shall become vacant, to reappoint competent persons to fill the same. One of the three appointed on each Board shall be designated as the Executive Inspector. Such officers, for the purposes of this Act, shall possess all the powers, rights, privileges, and immunities of customs officers, or officers acting under the Board of Health, and it shall be their duty to cause the various quarantine stations to be kept clean and properly fitted for use.

SEC. 3. The master of any vessel on which there shall have been shipped live animals for any port in this Kingdom shall, immediately upon arrival, notify the customs officers of such fact, and said officers shall at once cause the inspecting officers to be notified, and shall not permit the animals to be taken from the wharf or landing, nor any portion of the food or water, nor any effects connected therewith or provided for their use during the voyage, to be removed from the wharf or landing until the inspecting officer shall have inspected and passed the same.

SEC. 4. All live animals, except such birds and small animals as shall be specially exempted by the inspecting officer, shall be subject, on arrival in this Kingdom from any foreign port or country, to be quarantined at the expense of the owner or consignee thereof, in such places as shall be appointed by the Minister of the Interior, for a period of not less than thirty days, and for such longer period as shall be deemed necessary by the Board of Inspectors, on account of the presence of any contagious disease or distemper, or because the port or country whence such animals are brought is affected with such disease or distemper, or for any other good and sufficient reason having reference to the public good. Whenever, after careful examination and attention, the inspecting officer shall find that such animal or animals are infected with any disease or distemper of a nature dangerous to the live-stock of the country, he shall report the same to the Board of Inspectors, and if the majority of the Board of Inspectors shall decide that the public interests require, they shall cause such animal to be utterly destroyed ; said Board of Inspectors may also cause all the food and other effects connected with such animals, independently of the animals themselves, to be destroyed.

SEC. 5. Live animals passing between the different islands of the Kingdom may be quarantined, as set forth in Section 3, either at the port of shipment or delivery, on good cause shown to the inspecting officer of the port of entry nearest to the port of shipment or delivery.

SEC. 6. The Minister of the Interior, notwithstanding anything in this Act, may, from time to time, by proclamation declaring any port or country to be infected, absolutely prohibit the introduction of any animals therefrom until the restriction be removed.

SEC. 7. All imported animals, fodder, fittings, or effects landed contrary to the provisions of this Act, or taken or removed from quarantine until duly discharged, shall be forfeited to the use of the Hawaiian Government; and all animals brought into such quarantine grounds, or placed with any animals under quarantine, shall be deemed to come under the provisions hereof, and shall be subject to all the conditions of the same.

SEC 7 *a*. It shall be the duty of every person to report immediately to the nearest Executive Inspector or inspecting officer any animal on or about his own premises, or the premises of another, which he shall have reason to believe to be affected with any infectious or contagious disease or distemper, under a penalty of not less than five nor more than one hundred dollars for each offence.

SEC. 7 *b*. Said inspecting officer shall have the power to enter upon any premises where they have reason to believe there is any animal affected with any infectious or contagious disease or distemper of a nature dangerous to the live-stock of the country, and to cause any such animal to be placed in quarantine for such time as said officer may deem necessary; and shall have the power, with the approval of the majority of said Board, to cause any such animal to be destroyed.

SEC. 8. Any and all persons knowingly and wilfully violating any of the provisions of this Act, or assisting in so doing, or who shall purchase, take, or carry away any animals, fodder, effects or fittings connected therewith, before the same shall have been discharged by the inspecting officer to perform his duty, then such person or persons shall be liable to imprisonment at hard labor for any period not over six months or to a fine not over five hundred dollars, or both; and all such offenses may be tried before any police or district magistrate.

SEC. 9. There shall be collected from the owner or consignee of animals inspected under this Act one dollar per head for all horses, mules, and cattle; fifty cents per head for all sheep and goats; ten cents each for every other animal or bird; and when, from the nature of the case, the making of such inspection shall be unusually onerous or severe, 25 per cent. additional shall be paid to the inspector.

"All fees collected shall belong to the officer making the inspection, and shall be full compensation for his services for such inspection."

SEC. 9 *a*. The several executive inspecting officers of the Kingdom shall keep regular records of the proceedings of their respective Boards, and shall semi-annually make a full and detailed report of their transactions, including an account of their receipts and expenses, to the Minister of the Interior, who shall lay the same before the Legislature.

SEC. 9 *b*. All reasonable expenses incurred in placing any disesed animals in quarantine, and of feeding and caring for the same, including medical treatment while in quarantine, shall be paid by the owner or consignee of such animals.

Any executive inspector appointed under this Act may sue in his own name, or the majority of any of said Boards may sue in the name of such Board any such owner or consignee who shall refuse or neglect to pay the fees or expenses mentioned in this Act; or may in his or their discretion hold any animal for which the fees and expenses have not been paid after demand, and after five days' public notice sell the same at public auction (provided such animal be not affected with any infectious or contagious disease or distemper), and from the proceeds of such sale the executive inspector or majority of such Board may retain a sufficient amount to cover the fees and all expenses incurred, and the balance pay over to the owner or consignee of the animal thus sold.

SEC. 10. The Minister of Interior shall, from time to time, make and publish such rules and regulations as shall be necessary for the more efficient carrying into effect the provisions of this Act.

*Regulation of the Board of Health in Regard to Tuberculosis in Neat-cattle.*

SECTION 1. The presence of tuberculosis in any animal, the flesh of which is likely to be used as food or from which milk is obtained for use or sale, is hereby declared to be a cause of sickness and a menace to public health and safety.

SEC. 2. All persons who shall have in their possession any neat-stock shall, when requested to do so by an inspector of the Board of Health, permit such animal to be examined for the purpose of determining whether or not it is affected with tuberculosis, and allow all necessary experiments or operations to be made or performed for such purpose.

SEC. 3. A suitable number of inspectors of the Board of Health, for the purpose of examining all neat-stock suspected of being infected with tuberculosis may be appointed by the President of the Board when so authorized by the Board.

SEC. 4. It shall be the duty of such inspectors to examine all stock suspected of being infected with tuberculosis, and to make such experiments or perform such operations as may be necessary to determine whether or not the animal is so infected.

SEC. 5. All animals which shall show symptoms of tuberculosis shall be condemned by such inspector, and destroyed; and no part of the carcass of such animal shall be used for food or disposed of in any other manner likely to endanger public health.

SEC. 6. In any case of doubt the inspector shall forbid the further use or sale of the milk of said animal, and shall cause it to be kept separate from other non-infected animals; and, before condemning such animal, shall summon for consultation with him one or more of his fellow inspectors, and their decision in the case shall be final.

SEC. 7. The several inspectors of animals shall keep a record of all animals inspected by them, and their decision regarding the same, and shall make weekly reports of their doings to the Board of Health.

SEC. 8. Said inspectors of animals shall receive such pay as shall be voted by the Board of Health.

SEC. 9. These regulations shall take effect from and after the 15th day of April, A. D., 1897.

MILK.—*Fee.*

SECTION 80. The annual fee for a license to sell milk in the District of Honolulu shall be twenty-five dollars; for the town of

Hilo, which, for the purposes of this Act, shall be limited to a circle the radius of which shall be two miles from the Court House, shall be fifteen dollars; and for all other districts, five dollars.

*Penalty for adulteration, etc.*

SECTION 81. Any person who shall sell, or offer for sale, any milk which has been adulterated by the addition of water or other substance, or from which the cream has been skimmed or separated, unless the same is specifically and openly stated to be skimmed milk, shall be fined not more than fifty dollars.

*Inspection and confiscation.*

SECTION 82. Any police officer or agent of the Board of Health shall have power to inspect and test any milk sold, or offered for sale, and to confiscate any adulterated milk which he may find.

*Miscellaneous.*

Any bad or badly-cured meat, or any substance which is emitting a noxious, deleterious effluvium, must be immediately removed at the expense of the owner from the premises where it is stored, notice having first been given by the Board of Health or its agent.

No person shall keep swine in the city of Honolulu within one mile of the Post-office.

No person shall throw dead animals on any of the public streets or highways within one mile of the Post-office.

No slaughter-house will be allowed in the city of Honolulu within one mile of the Post-office, or on any of the public highways leading to the city, or on the banks of any river or stream the water of which is used for drinking or domestic purposes, or in any situation not first approved by the Board of Health or its authorized agent. (Section 285, Comp. Laws.)

No person will be allowed to dry or salt hides or skins or to store the same within the boundaries of the city of Honolulu, or in any place adjacent to or to windward of the city, or in any place not first approved by the Board of Health or its authorized agents.

*Slaughter-houses.*

No slaughter-house shall be maintained in any part of this Kingdom, or in any place where the Board of Health shall now or hereafter forbid the maintenance of the same.

### TO REGULATE THE PRACTICE OF DENTISTRY IN THE HAWAIIAN KINGDOM.

From and after the passage of this Act it shall be unlawful for any person or persons to practice dentistry in the Hawaiian Kingdom except upon a certificate issued from the Board of Dental Examiners.

*License is required.*

No person shall practice medicine or surgery as a profession in the Hawaiian Islands, either gratuitously or for pay, or shall offer to so practice, or shall advertise or announce himself, either publicly or privately, as prepared or qualified to so practice, without having first obtained from the Minister of the Interior, under seal of his department, a license in form and manner substantially as hereinafter set forth. Such license shall only be granted upon the written recommendations of the Board of Health.

